# The impact of the creative performance of agricultural heritage systems on tourists’ cultural identity: A dual perspective of knowledge transfer and novelty perception

**DOI:** 10.3389/fpsyg.2022.968820

**Published:** 2022-09-02

**Authors:** Huiqi Song, Pengwei Chen, Shuning Zhang, Youcheng Chen, Weiwei Zhao

**Affiliations:** ^1^Anxi College of Tea Science, Fujian Agriculture and Forestry University, Quanzhou, Fujian, China; ^2^Research Center of Tourism and Hospitality Management, College of Tourism, Huaqiao University, Quanzhou, Fujian, China; ^3^College of Art and Design, Yunnan University, Kunming, Yunnan, China

**Keywords:** Globally Important Agricultural Heritage Systems, creative performance, cultural identity, knowledge transfer, novelty perception, GIAHS, agricultural heritage tourism

## Abstract

Tourism in the Globally Important Agricultural Heritage System (GIAHS) is critical to the inheritance and innovation of excellent traditional farming cultures. Based on social identity theory, this paper explored the process by which agricultural heritage systems’ creative performance influences tourists’ cultural identity through 406 questionnaires from Chinese tourists. The results indicate that creative performance affects tourists’ cultural identity through a dual perspective of knowledge transfer and novelty perception. Furthermore, perceived authenticity acts as a moderator, weakening the impact of creative performance on tourists’ knowledge transfer, while perceived authenticity does not affect the process of tourists’ novelty perception. This research provides a fresh perspective on the sustainable development of agricultural heritage tourism. Meanwhile, it offers theoretical foundations and practical inspirations for the development of agricultural heritage’s creative tourism.

## Introduction

Heritage tourism has received extensive attention from all walks of life in recent years (Poria and Ashworth, 2009; Park et al., 2019). Agricultural heritage tourism is crucial to the inheritance and innovation of excellent traditional farming culture. On the premise of fully respecting agricultural traditions and cultural diversity of heritage sites, agricultural heritage tourism not only raises public awareness of cultural roots but also enhances the public’s sense of cultural pride and identity. Meanwhile, it promotes the cultural revival of agricultural heritage sites (Sun et al., 2013; Su et al., 2020). However, agricultural heritage tourism has faced unprecedented challenges in current practice processes, including the marginalization of traditional agricultural culture practices, insufficient expressions of traditional cultural values, and untapped dilemmas of potential for heritage awareness and local identity (Su et al., 2019, 2020). Therefore, it is urgent to conduct in-depth discussions on agricultural heritage tourism.

Agricultural heritage tourism is an indispensable part of heritage tourism, but there is little related research. Previous studies were limited to resource developments, protections (Sun et al., 2011), and development strategies (Sun et al., 2013) of agricultural heritage tourism. In recent years, scholars have mainly focused on community developments (Su et al., 2019), value analyses (Su et al., 2020), cultural management (Kajihara et al., 2018), traditional landscape preferences (Santoro et al., 2020), and stakeholders (Zhang and Smith, 2019). However, current research on agricultural heritage tourism mainly concentrates on development approaches and residents’ attitudes. They lack a discussion of tourists’ attitudes and behavioral intentions toward agricultural heritage tourism from a cultural perspective. Meanwhile, they also lack empirical research on tourists’ cultural identity in heritage sites from a micro perspective. Therefore, this study aims to make up for the deficiencies of current research.

Previous studies on the cultural identity of agricultural heritage included rural gastronomic heritage (Wondirad et al., 2021), cultural landscapes (Shen and Chou, 2021), ecosystems (Zhang et al., 2019b), and benefit costs (Wang et al., 2021). These studies mainly discussed the formation of cultural identity from the perspectives of residents and governments, and they usually used case studies or qualitative comparison methods. However, they rarely carried out quantitative research from the perspective of creativity. With the rise of cultural and creative tourism, creativity with more novel ways of expressing culture has extended to many traditional cultural fields (Blapp and Mitas, 2017). Creative performances help enhance tourists’ cultural perceptions (Richards, 2000). However, agricultural heritage tourism is still mainly about landscapes, and it has serious problems such as form simplifications and product homogeneities (Su et al., 2019). Therefore, creative performance as a breakthrough point can help open theoretical black boxes of tourist cultural identity formation in the process of agricultural heritage tourism.

How creative performances of Globally Important Agricultural Heritage Systems (GIAHS) affect tourists’ cultural identity is a pivotal question addressed in this study. Social identity theory provides a theoretical basis for cultural identity studies in tourism contexts (Pilving et al., 2021). The theory emphasizes that a process of social comparison provides stimuli for in-groups with similar characteristics to facilitate their transition from perception to identity (Stets and Burke, 2000). Based on this, creative performance is a stimulus effect. First, from the perspective of knowledge transfer, cultural and creative tourism provides tourists with opportunities for cultural learning. It deepens tourists’ understanding of a common culture and thus enhances their psychological identity (Huang and Liu, 2018). Second, in terms of a novel perception perspective, heritage tourism elements are perceived by tourists in a novel way and awaken their pleasures. They are consolidated through continuous exposure to cultural heritage and eventually form an individual cultural identity (Remoaldo et al., 2020). Finally, perceived authenticity motivates tourists to engage in creative tourism. It facilitates tourists’ perceived novelty and influences the degree of knowledge transfer to cultural heritage (Park et al., 2019). Accordingly, we infer that the factors influencing the creative performance of agricultural heritage on tourists’ cultural identity include novelty perception, knowledge transfer, and perceived authenticity (Zhang et al., 2021).

**Figure 1:**
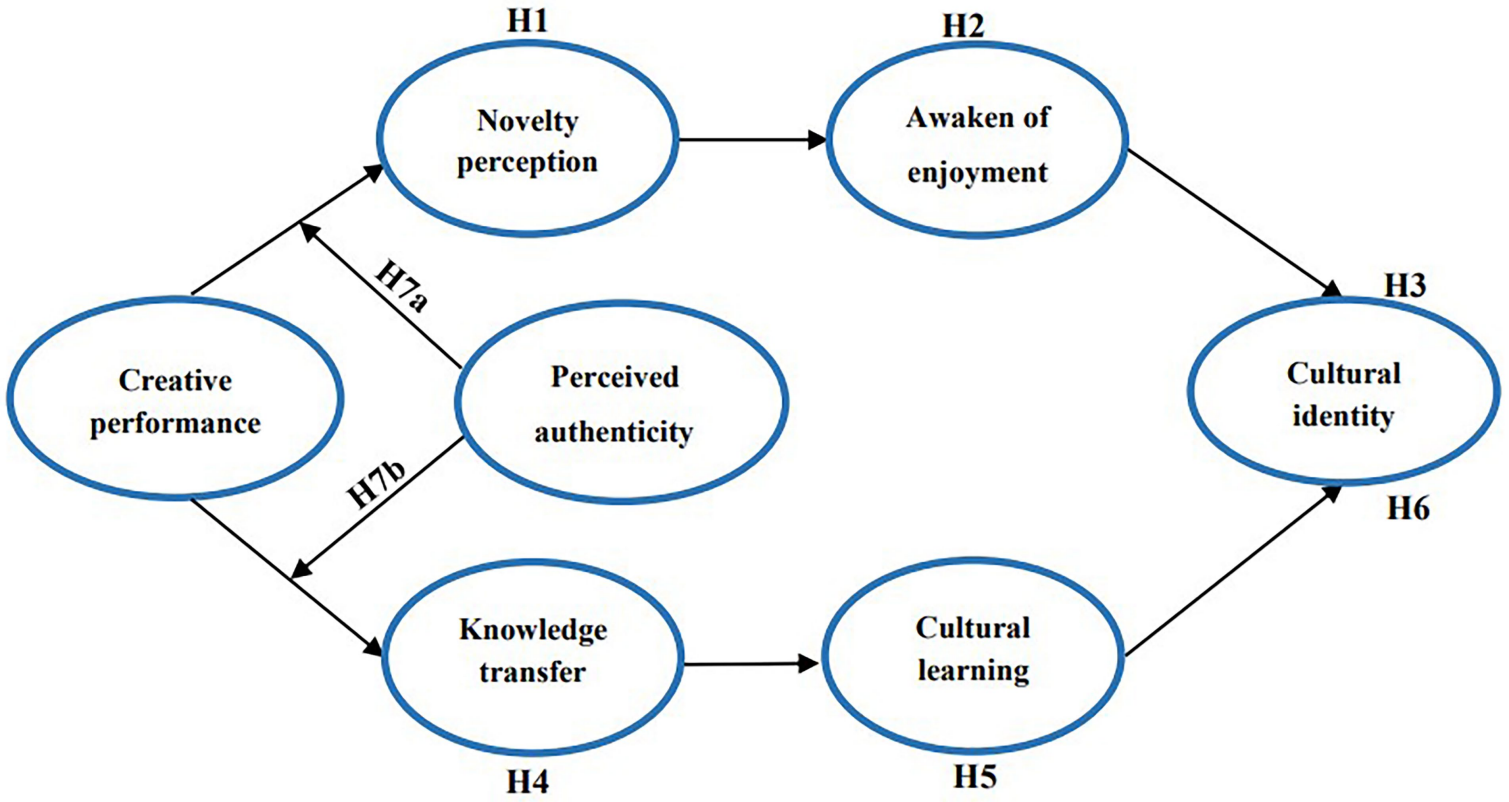
Research framework—hypothesized model.

Based on the above questions, the objectives of this study include the following: (1) addressing a vital issue of how creative performance affects cultural identity; (2) exploring the role of novelty perception and knowledge transfer in the influence process of creative performance on cultural identity; and (3) analyzing whether perceived authenticity plays a regulating role. This study explores the impact of creative performance on cultural identity by establishing a chain mediation model in Figure 1. It not only bridges the research gap of the cultural identity of agricultural heritage tourists from the perspective of cultural creativity but also provides the antecedent theoretical path for the cultural identity of heritage. Moreover, it expands the applications of social identity theory (Tajfel, 1978), knowledge transfer (Cooper, 2006), and novel perception (Petrick, 2002) in the field of agricultural heritage tourism. More importantly, this study provides management ideas for an integrated application of creative tourism and agricultural heritage. Meanwhile, it is conducive to the sustainable development of agricultural heritage tourism.

## Theoretical background and hypothesis development

### Social identity theory

Social identity theory derives from an individual’s knowledge of membership in a social group and the values and emotional meanings associated with that membership (Tajfel, 1978). It reflects the central role of individuals in the group identity process and the unique attributes of individuals and involves three psychological stages: social categorization, social identification, and social comparison (Lee and Gretzel, 2014). In heritage tourism, there is a link between the core identity attributes of individuals and specific heritage sites they visit, and tourism is seen as a way of establishing, maintaining, and creating personal identity. Social identity theory lays a foundation for the sustainable development of heritage tourism (Pilving et al., 2021).

First, the social classification stage of this theory emphasizes the functional and hedonic values of tourism activities. On the one hand, agricultural heritage tourism endows heritage with the functions of products or services, and the social identity embodied in the functional value of this product or service becomes part of the personal concept of the heritage tourism tourist (Tajfel, 1978). Therefore, they reflect the behavior of tourists as a specific group in the tourism experience, as well as the functional values associated with the identity of the tourist group. And heritage creative products or services have practicality and help visitors to gain knowledge or information related to the heritage experience (Chang et al., 2016). The accumulation and consolidation of knowledge and information further attracts visitors to heritage activities, which are more likely to meet the learning needs of tourists regarding history and culture in the interaction process (Richards, 2000). On the other hand, in the current environment, the expression of creative elements of agricultural heritage will give visitors a sense of pride and self-esteem from the uniqueness of the group to which they belong as creators and sharers of the value of agricultural heritage sites. It will satisfy visitors’ inner expectations of heritage, thus realizing their own psychological commitment to a unique identity, generating emotional meaning, and inspiring subjective feelings such as novelty and pleasure (Remoaldo et al., 2020). At the same time, hidden cultural feelings also further improve tourists’ cognitive processes and defining abilities of self-concept (Liu, 2018). The above two processes highlight tourists’ perceptions of the function and hedonic values of agricultural heritage tourism in the social classification stage. Second, the social identification stage of the theory advocates that individuals identify themselves according to groups’ common characteristics (Brown, 2000), including cognition, emotion, and evaluation (Chen et al., 2019). After heritage creativities have aroused tourists’ interests, it creates the perception that tourists belong to a special group of agricultural heritage tourists and attracts them to a deeper experience of relevant tourism projects. Furthermore, diversified projects focusing on the farming experience will make visitors emotionally suggestive, further attract and satisfy tourists’ expectations for the uniqueness and novelty of agricultural heritage. It also arouses a high-level sense of enjoyment in tourists (So et al., 2020), stimulates their positive attitudes toward heritage tourism, generates positive or negative values about themselves in relation to their sense of belonging to a group of agricultural heritage tourism tourists and thus promotes their sense of identity with agricultural culture, which will be consolidated with further contact with cultural heritage (Oliveira et al., 2020). Therefore, this process reflects an evolution of the cognition, emotion, and evaluation stages in the social identification stage of this theory. Finally, the social comparison stage implies that people with similar characteristics are classified as internal groups, and thus, they form unique groups (Stets and Burke, 2000). As external stimuli, creative performance and perceived authenticity convey special cultural symbols to tourists, facilitating the formation of a cognitive process of social comparison between the internal and external groups of tourists in heritage sites and meeting tourists’ needs to acquire knowledge (Wang et al., 2020). Moreover, knowledge learning further reinforces the image of the visitor as an agricultural heritage tourist. By communicating to others that they belong to a social group of heritage sharers and participants, they demonstrate the difference between themselves and those who do not visit the local area after acquiring heritage knowledge. Meanwhile, it encourages tourists to share heritage knowledge and realize transformations from cultural cognition to cultural identity (Zhang et al., 2021). Therefore, core views of social identity theory support the relationship between the cultural identity of agricultural heritage and relevant elements such as creative performance, novelty perception, and knowledge transfer. It provides theoretical support for the theoretical model construction of this study.

### The mediating role of novelty perception and awakening of enjoyment

Novelty perception is determined by the degree of contrast between current perception and past experience, and novelty seeking is an essential factor in measuring differences in individual tourists’ experiences of different destinations (Petrick, 2002; Chark et al., 2020). The creative performance of agricultural heritage involves cultural performances with real agricultural landscapes, farming cultures, rural folklore, etc., as the main content, and it includes cultural expressions such as human stories and poetry (Wang and Netemeyer, 2004; Farsani et al., 2019). According to the social comparison phase of social identity theory, creative elements are considered as an external source of stimulation that will bring new experiences to visitors. Because in the process of creative heritage experience, visitors will enhance their perception of something new from the new experience, which is likely to refresh visitors’ previous potential impressions of heritage itself. Therefore, positive creative performances at heritage sites may further attract visitors to engage with novelty. Previous studies have shown that heritage creative elements stimulate an individual’s quest for novelty (Tan et al., 2014), and heritage appeals stem from novelty seekers’ pursuit of creativity and a high level of involvement in creative activities (Remoaldo et al., 2020). Thus, first, creative performances help attract tourists’ attention to novelties and stimulates their engagement. Second, when tourists perceive something new, it can, to a certain extent, meet their inner expectations, stimulate their interest in the destination, and generate positive psychological feelings. For example, cognitive psychology shows that high novelty helps increase visitors’ interest in a destination and helps them express their psychological feelings when they receive a new experience, including stimulations and surprises (Lee and Crompton, 1992; Skavronskaya et al., 2019). Finally, novelty and practicality are the most prominent aspects of heritages’ creative products (Chang et al., 2016). Agricultural heritage systems stimulate a sense of novelty in tourists in agricultural culture by conveying creative elements such as heritage legends and knowledge of farming cultures to tourists. Thus, it satisfies their expectations of novelty and stimulates a high level of pleasure. This process is mainly motivated by heritage sites’ nature and novelty experiences (Jopp et al., 2021). Therefore, the following hypothesis is proposed based on the above theories and literature.

*Hypothesis 1*: Novelty perception mediates the relationship between creative performance and awakening of enjoyment.

As a tourism-related intrinsic motivation, the awakening of enjoyment reflects a visitor’s perception of positive emotions such as joy and pleasure during tourism experiences (Tan and Yang, 2021). On the other hand, cultural identity relies on the internal intention of self-perception and is a process of integrating heritage into the self-concept (Pratt, 2005). Social identity theory emphasizes that the formation of individual identification with a group is based on the cognitive, affective, and evaluative stages of social identity (Chen et al., 2019). When visitors develop positive emotions about a particular environment, they may develop behavioral intentions related to the tourism experience. And when this positive emotion is sustained, there is an ongoing cultural resonance and connection with the heritage site. Especially in the field of tourism, the awakening of enjoyment reflects an individual’s emotional state toward a preferred environment (So et al., 2020). It acts as a mediating element between stimuli and behavioral intentions and makes it easier to build positive attitudes toward tourists’ experiences and create high levels of behavioral intentions. This process involves factors such as the identification and internalization of personality (Oliveira et al., 2020). In the creative tourism experience, consistent with the social identification stage of social identity theory, tourists’ perception of heritage novelty may induce positive emotions in tourists. And when the positive sentiment is sustained, it further attracts visitors to the culture in question. Ultimately, sustained cultural interaction creates a sense of cultural identification with heritage for visitors. In this study, novelties such as agricultural legends, natural scenery, and folklore serve as environmental stimuli that are antecedents to inducing key emotional states in tourists, creating opportunities for tourists to learn about local culture and deepening their interaction with cultural heritage (Zhang et al., 2020). Therefore, synthesizing the above theories, this study argues that agricultural heritage sites rely on the novelty and uniqueness of creative tourism to awaken tourists’ sense of enjoyment, stimulate their cultural resonance, change their attitudes and intentions toward heritage tourism, and finally, enhance their sense of identification with heritage culture. Based on this, the specific assumptions are as follows:

*Hypothesis 2*: Awakening of enjoyment mediates the relationship between novelty perception and tourists’ cultural identity.

*Hypothesis 3*: Novelty perception and awakening of enjoyment play a remote mediating role in the relationship between creative performance and cultural identity.

### The mediating role of knowledge transfer and cultural learning

Knowledge acquisition is an interactive process in which visitors share knowledge and ideas during their experiences. It represents the ultimate goal of information dissemination, and it culminates in the sublimation of experiences (Baggio and Cooper, 2010). Hardy et al. (2018) identified knowledge transfer as an essential indicator of intergroup membership, in which knowledge and ideas are shared to create tourism products and experiences. Furthermore, cultural learning is a crucial motivation for tourists to experience cultural differences and the functional benefits of travel (Richards, 2011). According to social identity theory, in the process of creative tourism experience, elements such as unique customs and traditions of heritage sites accelerate the cognitive process of forming social comparisons between internal and external groups of tourists. They satisfy tourists’ needs for heritage knowledge, and the accumulation of heritage knowledge further enables a transformation from cultural awareness to cultural identity. Recent studies have shown that knowledge transfer is a key facilitator for individuals to acquire cultural learning (Lin et al., 2021). Especially in the field of heritage tourism, the transfer of heritage tourism knowledge prompts visitors to share knowledge and disseminate information and ideas, and thus, they acquire heritage knowledge through cultural learning. Thus, knowledge transfer drives behavioral changes in tourists. Furthermore, in the field of creative tourism, Liu (2018) argued that creative performance is an essential element of cultural and creative tourism through which tourists can gain new knowledge offered by cultural heritage. On the one hand, when individuals experience heritage, they also communicate and exchange creativity to contribute to improving knowledge and skills among themselves (Wang and Netemeyer, 2004). On the other hand, creative performance helps tourists develop interests in the cultural heritage itself and thus share knowledge and ideas to promote innovation in tourism products and services in heritage sites. Meanwhile, it maintains the competitiveness of heritage tourism destinations (Raisi et al., 2020). In short, when tourists enjoy the creative experience of heritage tourism, it is easy to achieve a transfer of heritage knowledge, which helps enhance their own positivity to learn about the cultures in question. Therefore, the following hypothesis is proposed:

*Hypothesis 4*: Knowledge transfer mediates the relationship between creative performance and cultural learning.

Cultural learning advocates learning about cultural heritage preservation while creating a fantastic destination image for visitors (McKercher and Du Cros, 2002). It reflects tourists’ interest in consuming cultural heritage sites and represents an essential motivation for tourists’ reception of new cultures and tourism experiences (Funk and Bruun, 2007). In heritage tourism experiences tourists are often accompanied by learning about heritage culture, and the results of cultural learning lead to an evaluation of the destination experience. When cultural learning is accumulated and sustained, it will lead to a sense of identity with the destination. And this sense of identity further enhances the cultural learning of visitors and deepens the cultural connection between tourists and the destination, which ultimately leads to a cultural identity for the heritage destination. Specifically, visitors’ interest in heritage attracts them to engage in cultural heritage living experiences and ultimately gain the ability to learn new knowledge (Blapp and Mitas, 2017). Lin et al. (2021) further asserted that when heritage travel is embedded with cultural significance, the demands of tourists to benefit from knowledge inspire them to learn more about local cultures at a deeper level. Cultural learning relies on the conceptual representation of knowledge learning by tourists, which, in turn, leads to an evaluation of the destination image. Importantly, with the deepening of cultural learning, visitors are more likely to acquire a sense of cultural identity with heritages (Kranz and Goedderz, 2020). Overall, when heritage knowledge is realized through cultural heritage creative tourism, it attracts tourists to learn about heritage culture in depth, which, in turn, consolidates their cultural identity with agricultural heritage sites. Therefore, the research hypotheses are as follows:

*Hypothesis 5*: Cultural learning mediates the relationship between knowledge transfer and tourists’ cultural identity.

*Hypothesis 6*: Knowledge transfer and cultural learning mediate the relationship between creative performance and cultural identity.

### The moderating effect of perceived authenticity

Perceived authenticity is described as an exploration of a specific social context and “unspoiled, original, traditional” authentic cultural activities that effectively reflect the uniqueness and sense of the history of cultures in the region (MacCannell, 1973; Kolar and Zabkar, 2010). In addition, tourism and its environment can facilitate transient experiences inspired by perceived authenticity (Fu et al., 2017). According to social identity theory, perceived authenticity as a source of external stimuli that accelerates the degree to which visitors perceive cultural experiences by revealing the original elements of tourists (Zhang et al., 2020). With the development of cultural tourism, perceived authenticity has become a critical factor in reflecting the traditional cultural aspirations of tourists and attracting them (Chung et al., 2018). Specifically, the combination of creative performance and perceived authenticity contributes to the novelty and uniqueness of tourists’ experiences (Zhang et al., 2019a). While creative performance is the best way to convey authenticity conservation and heritage, the interaction between the two helps to enhance visitors’ knowledge, understanding of heritage sites and strengthen the emotional connection between tourists and heritage sites. In addition, perceived authenticity is the basis for the sustainable development of heritage tourism. Agricultural culture heritage shows authentic farming cultures, ancient villages, and other original cultural veins, which may interfere with tourists’ novel perception of traditional cultures’ unique charms and affect the interactive experiences of tourists with cultural heritage (Boyd, 2002; Lee, 2015). Therefore, this study argues that the interaction of authenticity and creative performance can promote novel perceptions of heritage for visitors. And the following hypothesis is proposed in this study.

*Hypothesis 7a*: Perceived authenticity moderates the relationship between creative performance and novelty perception.

Perceived authenticity reflects the attractiveness of tourists’ involvement in tourism experiences (Wang, 1999). Visitors’ perceptions of heritage sites’ perceived authenticity include assessments of tangible and intangible aspects (Yi et al., 2018). Especially in the field of heritage tourism, creative tourism enhances visitors’ access to heritage knowledge and deepens their connection to the history and culture of heritage sites (Özdemir and Seyitoğlu, 2017). Meanwhile, it creates opportunities for them to learn about cultures (Richards, 2011). In fact, tourists seek to expand their knowledge and thus unleash their creativity in the process of deep involvement in creative experiences, which shows the inextricable relationship between creative tourism and knowledge transfer. Perceived authenticity, by basing cultural encounters in tourism activities on authenticity, mobilizes visitors’ attention to enhance learning about authentic cultures and it reflects the connection between culture and the tourism experience (Chen and Rahman, 2018). Especially in heritage tourism sites, visitors who participate in creative tourism are likely to deepen their learning about the authentic culture of the heritage through an authentic perception of the site. Thus, the ultimate experience of complete engagement is obtained, which facilitates the transfer of personal knowledge. Moreover, in the context of cultural and creative tourism, the desire for learning is often accompanied by a desire for authenticity (Richards, 2000). As MacCannell (1973) emphasizes, cultural and heritage attractions become an obsession with experiential authenticity as visitors seek authentic experiences. And the pursuit of authenticity makes it likely that those in the creative tourism experience will promote a desire for culture and the transfer of knowledge of heritage to the visitor because of the pursuit of authenticity in the experience. In addition, perceived authenticity is the key to gaining a competitive advantage in creative tourism locations. As Wang et al. (2020) pointed out, creative tourism uses perceived authenticity as a competitive advantage that can satisfy tourists’ need to acquire knowledge, providing them with a memorable experience. However, while tourists gain creative experiences at cultural heritage sites, perceived authenticity may also be used as a judgment criterion, and tourists are resistant to it. Thus, it will affect the effective transfer of heritage knowledge. Based on the above theoretical assertion, visitors with perceived authenticity are more likely to show more positive knowledge transfer after a deep heritage creative experience. Therefore, the following hypothesis is posited.

*Hypothesis 7b*: Perceived authenticity moderates the correlation between creative performance and knowledge transfer.

## Methodology

### Sample and data collection

Fujian Province is one of China’s crucial cultural heritage conservation areas. It has a unique traditional lifestyle and cultural and historical landscapes (Liu, 2019). In recent years, Fujian Province has pursued cultural tourism integration and regional cultural expansion strategies, promoting the integration of diverse cultural and creative tourism in the region (Zhang et al., 2019b). Therefore, the data of this study were obtained from surveys of jasmine cultivation and tea culture systems in Fuzhou, Fujian, China; the Tie Guanyin tea culture system in Anxi, Fujian; and the white tea culture systems in Fuding, Fujian, and Youxi, Fujian. These four systems are all located in Fujian Province, and they are representative of the important tea agricultural heritage sites in China (Min and Zhang, 2019). Among them, Fuzhou Jasmine Tea Culture System and Anxi Tieguanyin Tea Culture System are Globally Important Agricultural Heritage Systems (GIAHS). Tea cultural tourism in Fujian Province attracts hundreds of groups and organizations each year for tea tourism, ecology, etc. (Shen and Chou, 2021). Therefore, the case is typical and representative.

First, after reviewing literature on creative performance, perceived authenticity, cultural learning, knowledge transfer, novelty perception, the awakening of enjoyment, cultural identity, and other related topics, we conducted a preliminary survey before the study. Our research questions and questionnaire design were discussed by stakeholders, and the original measurement items were screened out for internal evaluation. Second, we confirmed that the respondent was a visitor who visited the above four cultural heritage sites. Third, four research assistants were invited to conduct short-term training, such as clarifying the purpose of the survey and the rules of the survey and declaring that no personal privacy issues were involved. Fourth, through face-to-face interview, the respondents completed the questionnaires according to their true feelings. Furthermore, we explained any questions that were unclear to the participants and we asked the assistants to check the questionnaires. Fifth, the respondents were asked to fill in each questionnaire, and small gifts were distributed as thanks. Sixth, the questionnaire collection was carried out from December 2021 to March 2022. We distributed 500 questionnaires, and 463 (92.6%) were returned. We tested the validity of the collected questionnaires, and after eliminating 57 invalid questionnaires due to incomplete or entirely consistent responses, 406 questionnaires (81.2%) remained for data analysis. The descriptive statistics of the respondents are shown in Table 1.

**Table 1:** Background of participants (*N* = 406).

Items	Frequency	Percent	Items	Frequency	Percent
Gender			Master or Doctor	63	15.50%
Male	219	53.9%	Numbers of experiences		
Female	187	46.1%	Once	101	24.90%
Age			2–3 times	124	30.50%
21–30	117	28.80%	4-5times	64	15.80%
31–40	168	41.40%	6 times or over	117	28.80%
41–50	93	22.90%	Monthly income (¥)		
51 or over	28	6.90%	3,500 or below	54	13.30%
Education background			3,501–5,000	79	19.50%
Junior high school or below	35	8.60%	5,001–8,000	122	30%
Senior high school	118	29.10%	8,001–12,500	95	23.40%
Junior college	124	30.50%	12,501 or over	56	13.80%
University	66	16.30%			

### Measurement

In this study, the English questions were translated into Chinese using the double-blind back-translation method and independently translated into Chinese by two professional translators. In addition, two translators who specialize in tourism management were invited to perform the back translation (Zhang et al., 2019a) as a way to maintain consistency between the English scale and the Chinese questionnaire. The variables were measured on a seven-point Likert scale, with “1” representing total disagreement and “7” representing total agreement. Specifically, (1) Referring to Zhang et al. (2020), four items were used to measure creative performance, reflecting visitors’ perceptions of the creative elements of agricultural heritage. (2) Referring to Hung et al. (2019) three-item scale was used to measure how the visitors learn culturally. (3) Referring to Chen et al. (2009), three items were used to measure knowledge transfer, reflecting knowledge change levels among tourists in heritage tourism. (4) Referring to Ayeh et al. (2013), three items were used to measure the awakening of enjoyment to examine the mood changes of tourists during heritage tourism. (5) Three-item scale of Lu et al. (2015) was used to measure the extent to which the visitors perceived the heritage to be authentic. (6) Four items from Duman and Mattila (2005) were used to measure novelty perceptions, which reflect visitors’ perceptions of novelty at heritage sites. (7) Finally, the three-item Cultural Identity Scale by Zhang et al. (2021) was used to measure the tourists’ cultural identity with the heritage site. (8) We also used some demographically relevant variables, such as gender, age, education level, number of visits, and average monthly income. (Kranz and Goedderz, 2020; Zhang et al., 2020).

### Data analyses

The means, standard deviations, factor loadings, composite reliabilities (CRs), and average variances extracted (AVEs) of each item are depicted in Table 2. As shown in Table 2, the values of Cronbach’s alpha values were all in the range of 0.800–0.896, indicating high internal consistency (Ayeh et al., 2013). In addition, the standardized factor loadings were above 0.644 (above 0.60) for each item in Table 2. AVE and CR were used to assess the reliability and validity, with CR values greater than 0.7 and AVE values greater than 0.5. They indicated the reliability and validity of the structure of this study (Liu, 2018). Table 3 depicts the means, standard deviations, correlations, and square roots of the mean squared deviations for each conformation. The correlations for all conformational surfaces were smaller than the square root of the mean explained variance. Thus, there was distinctive reliability and validity between the dimensions. To further measure whether the variables were highly correlated and thus there was a collinearity problem, this study tested the values of the variance inflation factor (VIF) of the independent and dependent variables. The results showed that all VIF values were less than 2.306 (<5.0); (Zhang et al., 2019b). The results showed that there was no collinearity in the study variables. In addition, this study strictly followed the appropriate steps to measure the issue of common methodological bias among variables using a well-established scale from an authoritative journal. In this study, Harman’s one-way test was used to calculate the loadings of all study indicators, and the first factor extracted explained only 38.12% of the variance, which was below the 50% threshold (Podsakoff and Organ, 1986). Therefore, there was no issue of common method bias.

**Table 2:** Descriptive statistics and confirmatory factor analysis.

Constructs and factors	Mean	SD	Factor loading	CR	AVE
Creative performance (Cronbach’s alpha = 0.896)				0.843	0.642
Agricultural Heritage Systems come up with novel ideas to meet tourist needs.	5.298	1.256	0.790		
Agricultural Heritage Systems generate multiple alternatives for tourist enjoyment.	5.475	1.210	0.845
Agricultural Heritage Systems provide new perspectives on performance.	5.552	1.181	0.767
Agricultural Heritage Systems pay attention to providing new methods to win tourists.	5.537	1.274	0.798
Cultural learning(Cronbach’s alpha = 0.849)				0.850	0.653
I feel that I am a member of the Agricultural Heritage Systems.	5.648	1.134	0.797		
I think the residents of the Agricultural Heritage Systems are my close friends.	5.692	1.109	0.790
I am similar to the inhabitants of the Agricultural Heritage Systems.	5.613	1.197	0.837
Knowledge transfer (Cronbach’s alpha = 0.832)				0.834	0.627
Knowledge related to agricultural heritage can enhance my cognitive ability of agricultural knowledge.	5.697	1.032	0.731		
Knowledge related to agricultural heritage can shorten my understanding of agricultural knowledge.	5.687	1.101	0.866
The Agricultural Heritage Systems can help me absorb relevant agricultural knowledge.	5.719	1.040	0.772
Awakening of enjoyment (Cronbach’s alpha = 0.877)				0.877	0.704
The Agricultural Heritage Systems make me feel happy.	5.761	1.016	0.856		
I am satisfied with Agricultural Heritage Systems.	5.793	0.997	0.820
It is pleasant to travel in Agricultural Heritage Systems.	5.862	1.033	0.840
Perceived authenticity (Cronbach’s alpha = 0.842)				0.897	0.687
Historical architectures are well preserved at Agricultural Heritage Systems.	5.498	1.113	0.886		
The Agricultural Heritage System is an authentic portrayal of ancient life and customs.	5.650	1.123	0.834
The Agricultural Heritage Systems present local history and culture very well.	5.877	1.047	0.793
Novelty perception (Cronbach’s alpha = 0.876)				0.880	0.670
Agricultural heritage tourism is a once-in-a-lifetime experience.	5.517	1.314	0.765		
The Agricultural Heritage Systems are unique.	5.948	1.129	0.755
Traveling in Agricultural Heritage Systems is a unique experience.	5.771	1.144	0.890
Traveling in an Agricultural Heritage System is a novelty.	5.608	1.238	0.801
Cultural identity (Cronbach’s alpha = 0.800)				0.815	0.599
I feel very proud to identify with Agricultural Heritage Systems.	5.998	1.017	0.864		
I consider it very important to maintain Agricultural Heritage Systems.	6.158	0.956	0.797
I feel very much that I am a part of Agricultural Heritage Systems.	5.768	1.211	0.644

**Table 3:** Means, standard deviations, correlations, and discriminant validity.

Variables	Mean	SD	1	2	3	4	5	6	7	VIF
1. Creative performance	5.466	1.074	**0.829**							
2. Cultural learning	5.651	1.006	0.706	**0.808**						2.093
3. Knowledge transfer	5.701	0.916	0.626	0.677	**0.792**					1.998
4. Awakening of enjoyment	5.805	0.909	0.739	0.664	0.650	**0.839**				2.158
5. Perceived authenticity	5.675	0.954	0.711	0.667	0.6 99	0.683	**0.801**			1.965
6. Novelty perception	5.711	1.032	0.604	0.603	0.569	0.653	0.529	**0.804**		1.696
7. Cultural identity	5.975	0.902	0.659	0.735	0.750	0.724	0.710	0.596	**0.774**	2.306

Correlations below the diagonal greater than 0.529 are significant at *P* < 0.001. The square root of average variance extraction is shown on the diagonal in bold.

### Confirmatory factor analyses

To test the individual construct factor loadings and check the structural validity, the adaptation index was used to make judgments. Since all variables were univariate, the overall model fit indicator was applied when performing the structural equation test (Bollen, 2014; Liu, 2018). The theoretically proposed seven-factor model showed a better fit (χ^2^ = 429.785, *p* < 0.001; χ^2^/df = 2.056; CFI = 0.963; IFI = 0.966; TLI = 0.956; NFI = 0.932; GFI = 0.912; AGFI = 0.884; RMSEA = 0.051). Therefore, it can be applied for further data analysis.

## Results

**Figure 2:**
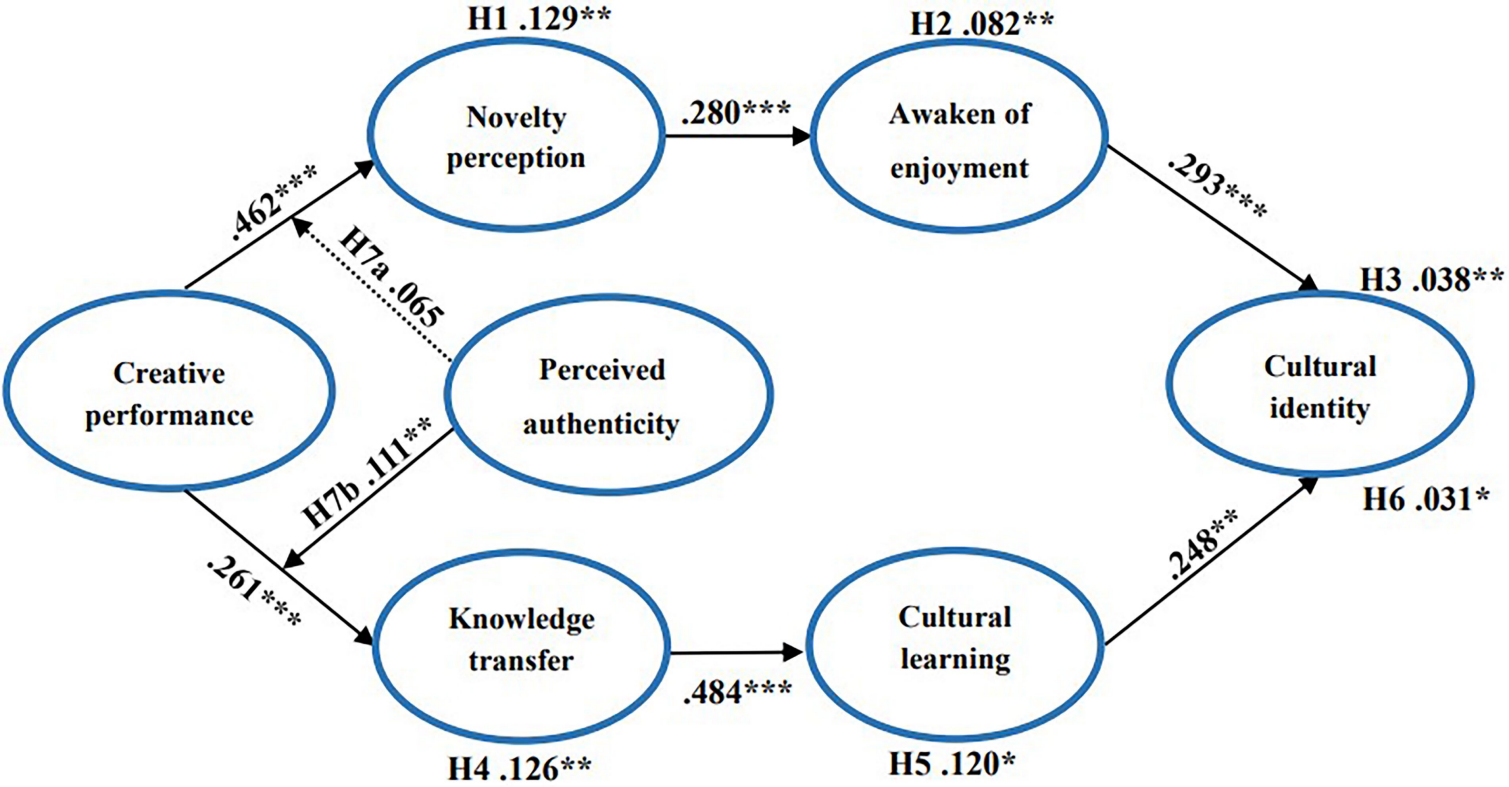
Research framework results. *N* = 406. *P < 0.05; **P < 0.01; ***P < 0.001.

First, this study used structural equation modeling (SEM) with AMOS 23.0 software to examine the mediating effects. The program had the bootstrapping method with 2,000 repetitions of sampling and the Monte Carlo approach to obtain 95% confidence intervals for bias correction (Liu, 2018) and produced unbiased estimates. Second, the moderating effect of perceived authenticity was tested using the hierarchical regression analysis of the macro program Process 3.3 in SPSS 24.0 software. As shown in Figure 2, the overall model fit is supported (χ2 = 302.921, *p* < 0.001; χ2/df = 1.396; CFI = 0.986; IFI = 0.986; TLI = 0.983; NFI = 0.952; GFI = 0.952; AGFI = 0.934; RMSEA = 0.031).

First, creative performance has a significant positive effect on novelty perception (β =0.462, *p* < 0.001). Novelty perception has a significant positive effect on the awakening of enjoyment, and awakening of enjoyment also has a significant positive effect on cultural identity. Their standardized regression coefficients were β = 0.280, *p* < 0.001; β = 0.293, *p* < 0.001. Hypotheses 1 and 2 test the mediating effects of novelty perception and awakening of enjoyment, respectively. As shown in Figure 2, novelty perception has a mediating effect between creative performance and awakening of enjoyment (β =0.129; *p* < 0.01). This result supports hypothesis 1. Awakening of enjoyment mediates the relationship between novelty perception and cultural identity (β =0.082; *p* < 0.01), supporting Hypothesis 2.

Second, Hypotheses 4 and 5 pertain to the mediating roles of knowledge transfer and cultural learning, respectively. Creative performance positively affects knowledge transfer, knowledge transfer positively affects cultural learning, and cultural learning positively affects cultural identity. The standardized regression coefficients were β = 0.261, *p* < 0.001; β = 0.484, *p* < 0.001; and β = 0.248, *p* < 0.01, respectively. Knowledge transfer mediated the relationship between creative performance and cultural learning (β =0.126; *p* < 0.01). Cultural learning mediated the relationship between knowledge transfer and cultural identity (β = 0.120; *p* < 0.05). Therefore, Hypotheses 4 and 5 are supported.

Third, the results of Hypotheses 3 and 6 indicate that “novelty perception → awakening of enjoyment” and “knowledge transfer → cultural learning” have a chain mediating effect on the relationship between creative performance and cultural identity (β =0.038; *p* < 0.01; β =0.031; *p* < 0.05, respectively, 0.031; *p* < 0.05). Table 4 shows that the 95% bootstrap confidence interval for the indirect effects does not contain zero. In conclusion, the mediating effects of this study are all supported.

**Table 4:** The results of mediating effect.

Hypothesis path	Standard error	Estimates	Bias-corrected 95% CI		Percentile 95% CI		Results
			Lower	Upper	Lower	Upper	
H1: Creative performance → Novelty perception → Awakening of enjoyment	0.040	0.129^**^	0.068	0.225	0.064	0.219	Support
H2: Novelty perception → Awakening of enjoyment → Cultural identity	0.034	0.082^**^	0.029	0.167	0.027	0.163	Support
H3: Creative performance → Novelty perception → Awakening of enjoyment → Cultural identity	0.017	0.038^**^	0.014	0.084	0.012	0.078	Support
H4: Creative performance → Knowledge transfer → Cultural learning	0.034	0.126^**^	0.070	0.206	0.066	0.200	Support
H5: Knowledge transfer → Cultural learning → Cultural identity	0.051	0.120^*^	0.037	0.239	0.025	0.224	Support
H6: Creative performance → Knowledge transfer → Cultural learning → Cultural identity	0.014	0.031^*^	0.010	0.067	0.007	0.062	Support

*N* = 406.

* *P* < 0.05;

** *P* < 0.01;

*** *P* < 0.001.

**Figure 3:**
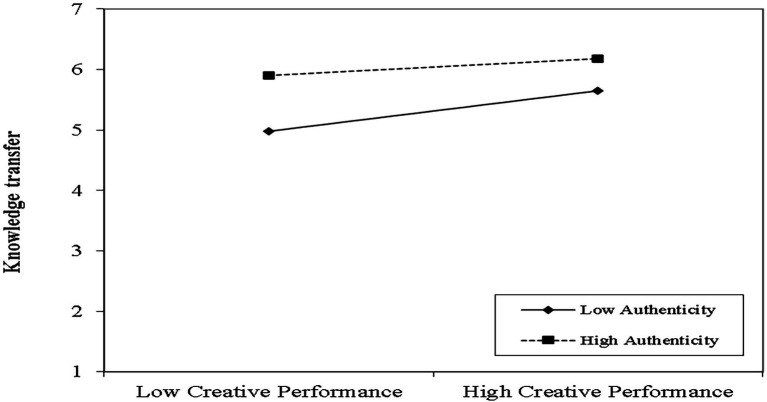
Interaction of creative performance and perceived authenticity on knowledge transfer.

Finally, Table 5 summarizes the moderating results for Hypotheses 7a and 7b. Models 1 and 3 represent only the control, independent, and moderating variables, while models 2 and 4 add interactions. The two-way interaction diagram of the regulatory effect is shown in Figure 3. The results show that the interaction between creative performance and perceived authenticity on visitors’ novelty perceptions is insignificant (β = −0.065; *p* > 0.1). Perceived authenticity did not moderate the relationship between creative performance and novelty perception, and Hypothesis 7a is rejected. In addition, the interaction between creative performance and perceived authenticity had a negative impact on knowledge transfer (β = −0.111; *p* < 0.01). As shown in Figure 3, the simple slope test indicates that as perceived authenticity increases, the positive relationship between creative performance and knowledge transfer diminishes. Therefore, Hypothesis 7b is supported.

**Table 5:** The results of moderating effect.

Dependent Variables	Knowledge transfer				Novelty perception			
	Model 1		Model 2		Model 3		Model 4	
	Coef.	t	Coef.	t	Coef.	t	Coef.	t
Interrupt	−0.088	−0.421	−0.026	−0.127	−0.280	−1.272	−0.244	−1.106
**Control variables**								
Gender	−0.009	−0.116	−0.027	−0.336	−0.048	−0.567	−0.059	−0.688
Age	0.016	0.338	0.008	0.173	0.029	0.581	0.025	0.489
Education	−0.001	−0.037	0.006	0.181	0.058	1.573	0.062	1.693
Number of experiences	0.011	0.306	0.015	0.434	−0.042	−1.113	−0.039	−1.046
Monthly income**(¥)**	0.012	0.337	0.011	0.323	0.056	1.527	0.055	1.521
Perceived authenticity	0.413	8.197^***^	0.378	7.389^***^	0.199	3.769^***^	0.179	3.304^**^
**Independent variable**								
Creative performance	0.280	5.573^***^	0.274	5.500^***^	0.435	8.224^***^	0.431	8.167^***^
**Interaction**								
Creative performance ^*^Perceived authenticity			−0.111	−3.119^**^			−0.065	−1.714
**Model statistics**								
R^2^	0.399		0.413		0.337		0.342	
R^2^_adj_	0.389		0.402		0.326		0.329	
F	121.533^***^		9.727^**^		94.463^***^		2.938	

*N* = 406.

* *P* < 0.05;

** *P* < 0.01;

*** *P* < 0.001.

## Conclusion and discussion

First, creative performances of agricultural heritage can enhance the cultural identity of tourists. This study supports that creative performance still plays a vital role in the behavioral intention of tourists in the context of agricultural cultural creative tourism. As Su et al. (2019) pointed out, heritage sites should stimulate stakeholders to engage in traditional agricultural activities through innovation to enhance heritage cultural vitality. Furthermore, the multiple chain mediators in this study follow the triggering mechanism of social identity theory (Tajfel, 1978). This is consistent with the findings of Sun et al. (2013) that in the context of agricultural heritage tourism, we can enhance tourists’ sense of identity by raising awareness of cultural roots and appreciation of traditional farming knowledge. Therefore, agricultural heritage in the context of cultural and creative tourism enhances tourists’ experiences at the psychological (perceiving traditional agricultural activities) and behavioral levels (learning traditional farming cultural knowledge) with the help of creative elements and forms tourists’ cultural identity.

Second, a high level of perceived authenticity did not moderate the relationship between creative performance and visitors’ novelty perception. First, the reason for this may be that due to the limited range of stakeholders, we currently consult with and the fact that the process may require more stakeholders to reach a mediation decision. Second, the result in the current scope of the study reflects that high perceived authenticity makes tourists pay more attention to the original cultural value of heritage, and the novelty of tourists’ feeling of agriculture cultural creativity then fades. As Su et al. (2019) pointed out, in a highly productive modern society, groups associated with Chinese agriculture cultural heritage sites are less emotionally and functionally attached to agriculture than their traditional ancestors. Therefore, when agricultural heritage shows high perceived authenticity based on the land, the creative elements that it contains do not effectively attract visitors to the novel perception of agricultural heritage.

Third, a high level of perceived authenticity has a negative moderating effect on heritage knowledge transfer. First, although research for tourists has covered as much ground as possible, current tourists have limited knowledge of heritage sites due to a short period of time. Therefore, more stakeholders need to be researched in the future to further deepen the findings. Second, to some extent, the results of this study reflect an impact of the paradoxical relationship between creative performance and perceived authenticity on changes in tourists’ knowledge transfer. The perceived authenticity of agricultural heritage involves authentic cultural expressions unique to agricultural heritage, which is one of the characteristics that distinguish agricultural heritage from other forms of heritage tourism. According to the regression results of authenticity in Table 5 and Figure 3, the higher the authenticity, the more negative the coefficient in Figure 3 (β = −0.111; *p* < 0.01) and the flatter the slope in Figure 3, reflecting the negative moderating effect of authenticity in the process of knowledge transfer by creative performance. Therefore, when the perceived authenticity of an agricultural heritage site is too high, in the scope of the current research, the creative performance of heritage tourism cannot positively influence local tourists’ knowledge acquisition. Specifically, when the perceived authenticity of the landscape of agricultural heritage sites is too high, crop-based agricultural heritage sites are vulnerable to natural risks and therefore suffer from the seasonality of tourism. It lacks adequate expressions of the value of agricultural heritage tourism (Su et al., 2020) and weakens the impact of creative tourism on knowledge transfer to tourists. When the perceived authenticity of agricultural heritage tourism is too low, creative tourism, as a novel method of promotion, is more likely to influence the process of agricultural heritage knowledge transfer to tourists.

## Theoretical contributions

The first contribution of this study is the construction of a theoretical path for the formation of tourists’ cultural identity in agricultural heritage sites based on a creative tourism perspective. Previous studies have shown that factors such as tourists’ emotions (Zhang et al., 2021), residents’ perceptions of benefits and costs (Wang et al., 2021), and local cuisine (Wondirad et al., 2021) contribute to the formation of cultural identity in heritage tourism. However, few studies have explored the antecedents of the cultural identity of agricultural heritage in the context of creative tourism. Furthermore, although some studies have focused on the role of creative performance in the cultural development of rural areas (Blapp and Mitas, 2017), they imply the attractiveness of creative tourism in agricultural heritage to tourists (Farsani et al., 2019) but have not empirically explored the influence mechanism of creative performance on cultural identity, and this paper fills this research gap. Importantly, this study extends Liu’s (2019) findings that creative performance in the context of traditional Chinese culture influences tourists’ behavioral intentions. This study makes a breakthrough by using multiple mediating and moderating mechanisms to emphasize the guiding role of creative performance in promoting the cultural identity of agricultural heritage tourists. In addition, this process is consistent with the social comparison process of social identity theory, which verifies the reliability of its path implementation (Stets and Burke, 2000). Finally, for the first time in agriculture heritage tourism, we explore the relationship between creative performance and cultural identity. The results not only enrich the research field of creative perspectives but also fill the gap between the integration of tourist identity issues in the field of GIAHS and the field of creative tourism.

The second contribution of this study is its clarifying that the theoretical path of creative performance on tourists’ cultural identity is a process related to psychological and behavioral mechanisms. Although many factors, such as cultural memory and cultural learning (Zhang et al., 2021), contribute to the formation of combined traces of cultural identity in the field of cultural and creative tourism, few studies have explored the mechanisms mediating the relationship between creative performance and cultural identity in terms of tourists’ psychological factors (novelty perception) and behavioral factors (knowledge transfer) separately. This study extends the research on consumer psychology and organizational behaviors (Light, 2017). In addition, the study results extend the research related to creative tourism of agricultural heritage. Farsani et al. (2019) elucidated tourists’ attitudes toward creative tourism in agriculture heritage. However, unlike previous studies, this study focuses on theoretical pathways rather than causal discussions of a single variable (Wondirad et al., 2021). It emphasizes the complex path composition of the impact of the creative performance of agricultural heritage on the cultural identity of visitors. Meanwhile, the multiple mediation-regulation mechanisms of cultural tourism, creative experience, and tourists’ behavioral intention from Huang and Liu (2018) were applied in the field of agricultural cultural heritage tourism in a breakthrough. In addition, Zhang et al. (2021) found that cultural learning positively impacts cultural identity. This study further emphasizes that the influence of cultural learning on cultural identity is caused by knowledge transfer and creative performance as preceding factors. It provides a reference for exploring the relationship between psychological factors and the cultural identity of tourists. More importantly, social categorization and social identification stages in social identity theory are consistent with this process, which provides theoretical support for this study (Chen et al., 2019).

Finally, the findings uncover the differential impact of perceived authenticity on the knowledge transfer of traditional farming culture in the creative tourism of agricultural heritage. Previous studies have shown that perceived authenticity positively affects visitors’ knowledge transfer behavior (Wang et al., 2020), and it has a positive moderating effect between creative performance and changes in the cultural knowledge of visitors (Aldebert et al., 2011; Zhang et al., 2020). However, few studies have demonstrated the impeding factors of perceived authenticity in the role of creativity and knowledge transfer, and few studies have applied perceived authenticity to empirical research in the field of agricultural heritage tourism. However, this study emphasizes perceived authenticity as an important factor hindering the impact of creative performance on tourists’ knowledge transfer, creatively extending perceived authenticity research to the field of agricultural heritage tourism and providing ideas for the future development of agricultural heritage cultural education. More importantly, the results validate Min and Zhang’s (2019) views on balancing tradition and innovation in GIAHS and support the moderate development of creative tourism in GIAHS. Finally, the study complements the three processes of social identity theory. It highlights the hindering effect of authenticity on stimulus effect in the process of social comparison (Stets and Burke, 2000), and provides a new research perspective for further research on cultural identity of agricultural heritage tourism.

## Management implications

While the range of stakeholders consulted in this study was limited, and management responses of different stakeholders may vary by context and level of knowledge, future engagement processes may require additional stakeholders to reach mediated decisions. However, for the current scope of the research, this study provides the following implications for the development and management of agricultural and cultural heritage sites. First, managers of agricultural heritage sites should clarify that the formation of tourists’ cultural identity requires an enhanced sense of creative experience in heritage tourism. It requires the governments and managers of agricultural heritage sites to take creative performance as the starting point to create a cultural and creative tourism system for agricultural heritage. For example, the government should advocate attracting tourists through traditional cuisine and farming civilization unique to the heritage site to create products and services for tourists of heritage sites with creativity (Farsani et al., 2019). Managers should also develop forms of creative tourism tailored to specific agricultural heritage sites. For example, for the rice-type agricultural heritage systems, managers should develop cultural and creative services and products in the Rice-fish agricultural system (RFAS) and the Rice-fish-duck agricultural system (RFDAS). The development of creative forms of tea-based agricultural heritage, such as creative tea estates and tea cultural and creative products, can help enhance the understanding of tourists’ identity with heritage sites (Shen and Chou, 2021).

Second, it is necessary to improve tourists’ heritage and cultural identity with the help of psychological and behavioral factors. The management of heritage sites needs to allocate available resources wisely so that they could improve tourists’ awareness and knowledge education levels about agricultural heritage. Meanwhile, they could enhance the heritage and protection of important cultural values of agricultural heritage by tourists (Liu, 2019). Examples of such applications include exhibitions of knowledge of farming cultures and ancient legends; the promotion of the integration of agricultural heritage with new technologies to enhance tourists’ perception of cultural values of heritage; the development of visual heritage tourism to awaken the interests of foreign tourists (Zhang et al., 2020); and the appeal to local tourists to preserve and pass on heritage to evoke a sense of mission (Funk and Bruun, 2007). In addition, managers should pay attention to the dynamic development of cultures of agricultural heritage sites and integrate them as closely as possible with cultural and educational activities. For example, developing cultural landscapes or cultural and creative products based on agricultural history and creating educational lectures on agricultural heritage cultures (Farsani et al., 2019) can promote the heritage and development of agricultural heritage creative tourism. In addition, cultural scenes are displayed through natural scenery to educate and entertain, and the artistic atmosphere is reinforced by idyllic rural scenery (Jopp et al., 2021). For example, local scenic spots guide tourists to learn deeply about farming culture through the natural scenery and topography of heritage sites, which, in turn, inspire them to explore the cultural and educational value of heritage and enhance their local identity with it.

Third, managers need to realize that enhancing the risk management of agricultural heritage sites is critical to the cultural experiences of visitors. Studies have shown that agricultural heritage sites suffer from tourism seasonality and that too high perceived authenticity can prevent the full expression of cultural tourism in heritage sites. Therefore, heritage site managers can use a combination of static and dynamic approaches to avoid risks for agricultural landscapes vulnerable to natural factors. For example, the construction of agrarian museums such as agricultural technology and farming culture and static forms such as the opening of creative B&Bs in traditional villages (Sun et al., 2011) help tourists shift their attention to heritage-derived products and services when they are unable to perceive agricultural heritage dynamically due to seasonality. In addition, the risk management of agricultural heritage inheritors should be strengthened to avoid the risk of losing agricultural heritage skills and to promote a deeper understanding of agricultural culture among tourists (Min and Zhang, 2019). For example, they could hold master competitions to screen quality farming skills in visitor experiences and offer creative programs around female inheritors to enhance risk management for inheritors. Meanwhile, they could increase visitors’ awareness of the value of heritage transmission.

## Limitations and recommendations for future research

Although this study proposes and extends thinking related to agricultural heritage’s cultural identity and creative tourism (Farsani et al., 2019), there are still some limitations. First, because the background of the participants and their knowledge or perception of tradition and authenticity may be limited, the scope of stakeholder research is still limited, although a certain degree of pre-research is conducted to try to facilitate prior agreement among stakeholders on the knowledge of relevant elements, etc. Therefore, future research processes may require additional stakeholders to reach mediation decisions or management insights. Second, this study demonstrates the critical role of the creative performance of agricultural heritage on tourists’ cultural identity. However, tourists’ cultural identity with agricultural heritage may also be influenced by other emotional or cognitive factors, such as place attachment (Zhang and Smith, 2019), value perceptions (Su et al., 2020), and community livelihoods (Sun et al., 2011). Therefore, future research should determine the influences of emotional and value perception factors of interactions with residents or individual characteristics on tourists’ cultural identity. Third, this study verifies the impact of creative performance on cultural identity from tourists’ perspectives regarding novelty perception and knowledge transfer. However, the relevant conclusions are based on questionnaire surveys and structural equation modeling analyses. Although this study used multiple mediating-regulatory mechanisms to clarify the antecedent paths of cultural identity further, the robustness of the findings should be further explored in the future using qualitative interviews (Sun et al., 2013). Alternatively, we should explore the theoretical path of cultural identity based on residents’ perspectives to improve the study of stakeholders’ attitudes toward agricultural heritage tourism (Su et al., 2020; Wang et al., 2021). Finally, the use of cross-sectional surveys and self-reported data to examine the cascading mediating effects is a possible limitation of this study.

## Data availability statement

The original contributions presented in the study are included in the article/supplementary material, further inquiries can be directed to the corresponding authors.

## Author contributions

HS collected the data and wrote the first draft. SZ, YC, and PC provided valuable suggestions for the first draft. All authors contributed to the article and approved the submitted version.

## Funding

This study was supported by the project “National Social Science Foundation Temporal and spatial differentiation law 489 and management response of ecological tourism industry in China” (21BGL148), the project “Innovation Strategy Research 495 Program of Fujian Province, research on collective memory construction and living protection of 496 Fujian tea cultural heritage (2021R0039),” and the project “Excellent Master program of Fujian Agriculture and Forestry University, (1122YS01002).”

## Conflict of interest

The authors declare that the research was conducted in the absence of any commercial or financial relationships that could be construed as a potential conflict of interest.

## Publisher’s note

All claims expressed in this article are solely those of the authors and do not necessarily represent those of their affiliated organizations, or those of the publisher, the editors and the reviewers. Any product that may be evaluated in this article, or claim that may be made by its manufacturer, is not guaranteed or endorsed by the publisher.
